# Prospective Assessment of Risk Factors Influencing Facial Nerve Paresis in Patients after Surgery for Parotid Gland Tumors

**DOI:** 10.3390/medicina58121726

**Published:** 2022-11-25

**Authors:** Ewa Głuszkiewicz, Paweł Sowa, Maciej Zieliński, Monika Adamczyk-Sowa, Maciej Misiołek, Wojciech Ścierski

**Affiliations:** 1 Department and Clinic of Pediatric Neurology, The Independent Public Clinical Hospital no. 6 of the Medical University of Silesia in Katowice, John Paul II Upper Silesian Child Health Centre, 40-752 Katowice, Poland; 2 Department of Otorhinolaryngology and Oncological Laryngology in Zabrze, Faculty of Medical Sciences in Zabrze, Medical University of Silesia, 40-055 Katowice, Poland; 3 Department of Neurology in Zabrze, Faculty of Medical Sciences in Zabrze, Medical University of Silesia, 40-055 Katowice, Poland

**Keywords:** facial nerve paresis, parotidectomy, blink reflex, electroneurography

## Abstract

*Background and objectives:* Facial weakness is the most important complication of parotid gland tumor surgery. The aims of this study are as follows: (1) assessment of the prevalence of postparotidectomy facial nerve dysfunction; (2) clinical and electrophysiological assessment of the facial nerve function before parotidectomy and at 1 and 6 months postoperatively; (3) assessment of the association of postoperative facial palsy with selected risk factors; and (4) assessment of the correlation between the results of clinical and neurophysiological assessments of facial nerve function. *Materials and Methods*: This study comprised 50 patients (aged 24–75 years) who underwent parotidectomy at the Department of Otolaryngology and Laryngological Oncology in Zabrze, Poland between 2015 and 2017. The evaluation included neurological, clinical and electrophysiological assessments of the facial nerve prior to surgery and at 1 and 6 months postoperatively. *Results*: No facial palsy was found preoperatively or 6 months postoperatively. Facial nerve dysfunction was found in 74% of patients 1 month postoperatively. In most cases (54%), paresis was mild or moderate (House–Brackmann grades II and III). The results of electrophysiological tests before parotidectomy were either normal or showed some mild abnormalities. We found a statistically significant correlation between the clinical assessment of the facial nerve function (based on the House–Brackmann scale) one month postoperatively and the latency of the CMAP response from the orbicularis oculi and orbicularis oris muscles. In all three studies, a statistically significant correlation was found between the amplitude of the compound muscle action potential (CMAP) of the orbicularis oris muscle and the degree of facial nerve weakness. *Conclusions*: The factors that may influence the risk of postoperative facial nerve paralysis (prolonged surgical time and the size and location of the tumor other than in the superficial lobe only) may indirectly suggest that surgery-related difficulties and/or surgeon experience could be crucial to surgery safety.

## 1. Background

Despite the continuous development of diagnostic methods and surgical techniques, both the diagnosis and treatment of parotid gland tumors pose serious clinical and therapeutic problems. This is due to the complex anatomical conditions of this region; the high histological differentiation of this group of tumors; and the need for radical surgical treatment with the preservation of the facial nerve function. The paresis or paralysis of the facial nerve is the most important complication of parotidectomy. The literature data show that permanent facial nerve paralysis occurs with a prevalence of 0–7%, while transient facial nerve conduction disorder is found in 8% to 65% of patients who undergo surgery [1,2,3,4]. Different papers stress the influence of many risk factors for postoperative facial nerve palsy (FNP), such as location and the histological type of the tumor, the type and duration of surgery, intraoperative bleeding, electrocoagulation (a distance of <1.5 cm from the nerve) and the need for the dissection of nerve branches from the tumor [5,6,7].

Notably, the absence of the clinical features of FNP in patients with parotid gland tumors does not exclude the existence of subclinical changes. In the case of slow-growing tumors, the first clinical symptoms may occur when over 50% of active axons are damaged [1,8,9,10]. Considering the above reports, a thorough assessment of the facial nerve function before parotidectomy is an important part of preoperative management and a reference point for the further postoperative monitoring of the nerve function [1,9,10,11]. Salivary gland tumors are relatively rare and account for 3–10% of all head and neck neoplasms. In the United Kingdom, the estimated global incidence rate ranges between 0.4–13.5 cases per 100,000 annually [12]. Mortality rates due to salivary gland malignancies vary depending on the stage of the disease and the tumor type. An over 5-year survival rate is found in 72% of patients [12].

## 2. Objectives

The aim of this study was to create a prospective assessment of the prevalence of the paresis or paralysis of the facial nerve after parotidectomy. The facial nerve function was also assessed in patients preoperatively and 1 and 6 months postoperatively. The important element of the study was related to the analysis of the relationships between postoperative facial nerve injury and selected risk factors, including the size and location of the tumor; histological type; tumor-related complaints; systemic diseases; stimulants; alcohol abuse; concomitant neurological disorders, type, duration and course of surgery; intraoperative bleeding and electrocoagulation (a distance of <1.5 cm from the facial nerve); and the need for the dissection of nerve branches from the tumor. Moreover, the relationships between the results of the clinical and neurophysiological assessments of the facial nerve pre and postoperatively were analyzed.

## 3. Materials and Methods

This study involved 50 patients (aged 24–75 years), i.e., 34 (68%) women and 16 (32%) men, aged 24–75 years who underwent surgery between 2015 and 2017 at the Department of Otorhinolaryngology and Laryngological Oncology in Zabrze of the Medical University of Silesia in Katowice, Poland. Parotid gland tumors were diagnosed in all patients based on clinical and ultrasound examination and thin needle aspiration biopsy. The inclusion criteria were as follows: primary parotid gland tumors and primary surgery. The exclusion criteria were as follows: the recurrence of parotid tumors, a history of other salivary gland diseases, radio and/or chemotherapy in the head and neck cancers, tumors penetrating the parapharyngeal space from the salivary gland, central facial nerve paralysis and no written consent to participate in the study. Each patient underwent neurological examination and clinical and electrophysiological assessment of the facial nerve preoperatively and 1 month and 6 months postoperatively. Clinical evaluation of the facial nerve function was performed using the House–Brackmann scale (HB scale) [13]. Electrophysiological examination of the facial nerve included the assessment of nerve conduction using electroneurography (ENoG) and the blink reflex test.

Electrophysiological studies were conducted at the Department of Neurology of the Medical University of Silesia in Zabrze using a two-channel EMG device (Viking Nicolet Biomedical Inc., San Carlos, CA, USA).

- Electroneurography

The following parameters were assessed: the compound muscle action potential (CMAP) amplitude and standardized latency. The ratio of the CMAP amplitude on the symptomatic side compared to the asymptomatic side was analyzed and a result > 0.7 was considered normal. The range of 0.7–0.1 was considered the mean degree of damage, whereas a result < 0.1 was regarded as significant damage. Due to the high variability of the latency, the standardized latency, which is a more stable and objective parameter, was selected for the analysis. A standardized latency < 0.35 ms/cm was considered normal and a result of 0.35–0.70 ms/cm was regarded as the mean prolongation of standardized latency, whereas a result > 0.7 ms/cm was considered significant prolongation of standardized latency [14,15].

- Blink reflex

The parameters obtained during the blink reflex test included the latencies of R1, R2 and R2′ waves (expressed in ms). Latencies of R1 and R2 waves were analyzed during stimulation of the symptomatic side, whereas the latency of R2′ was assessed during stimulation of the asymptomatic side. The prolongation of the latency of these waves is related to facial nerve injury on the symptomatic side. The reference values of the latencies adopted in the study were 10 ms in the case of R1 waves and <30 ms for R2 and R2′ waves.

The obtained latency values of individual waves were compared to the reference values. Based on this comparison, the facial nerve function was assessed as normal (latency within the reference values), mild disorders (latency prolongation of 1–100%) and significant disorders (when the latency of individual waves was more than twice the reference values) [16].

- Statistical analysis

Statistical analyses were performed using IBM SPSS Statistics 23. The analyses of basic descriptive statistics, order regression, logistic regression and correlation with Kendall’s τ coefficient were conducted. The classical threshold of α = 0.05 was considered significant. However, a probability of the test statistics of 0.05 < *p* < 0.1 was interpreted as significant at the statistical trend level.

## 4. Results

The neurological assessment performed preoperatively and 6 months postoperatively showed that none of the patients presented with facial nerve dysfunction. No other injuries to the central or peripheral nervous system were found. However, based on a detailed neurological examination, postoperative assessment at 1 month revealed FNP of different severities in 37 (74%) patients. In most cases (17 patients; 34%), mild paresis (HB scale grade II) or moderate nerve function impairment (grade III; 10 patients–20%) was found. More severe paresis that resulted in significant discomfort in patients was found in a lower percentage of patients, i.e., grade IV in eight (16%) patients and grade V in two (4%). Histological findings confirmed pleomorphic adenoma in 23 (46%) patients and Warthin tumors in 20 (40%) patients. Other diagnoses were found in seven patients. The grades of paresis depending on tumor location are presented in Table 1.

Medium-size tumors (2–4 cm) were found in 50% of patients, tumors < 2 cm were observed in 9 (18%) patients and tumors > 4 cm were found in 15 (30%) patients.

Time between diagnosis and surgery ranged from 3 months to 20 years (mean 2 months, median 57.99 months). A two-fold increase in tumor size was observed at 1 month before surgery in four (8%) patients. Other factors affecting the condition of the peripheral nerves found in the study group included diabetes mellitus in four (8%) patients, polyneuropathy in two (4%), absorption disorders in two (4%) and regular intake of stimulants in six (12%) patients. No other neurological disorders were found except for polyneuropathy. Other complaints (mostly tumor-related pain) occurred in five (10%) patients.

In most cases, partial superficial parotidectomy was performed, i.e., in 38 (76%) patients. Superficial parotidectomy was performed in six (12%) patients, and total parotidectomy was also performed in six (12%) patients.

Clinical examinations performed one month after surgery showed that most patients who underwent partial superficial parotidectomy presented with normal facial nerve function or with mild paresis (HB scale grades I/II). With regard to superficial parotidectomy and total parotidectomy, the percentage of patients without facial nerve dysfunction or with mild paresis was lower than that of patients with higher grade paresis (Table 2). The duration of parotidectomy ranged from 60 to 180 min (mean 100 min; median 90 min). A small amount of intraoperative bleeding occurred in 12 (24%) patients, an average amount in 34 (68%) and a large amount in 4 (8%) patients. Electrocoagulation (of a distance < 1.5 cm from the facial nerve) was performed in most cases (35 patients; 70%). Tumors were wrapped by the branches of the facial nerve in 23 (52%) surgical procedures.

### 4.1. Electrophysiological Assessment of the Facial Nerve Function before Surgery and at 1 Month and 6 Months after Parotidectomy

#### 4.1.1. ENOG Examination before Parotidectomy

With regard to recording responses from the orbicularis oculi muscle, preoperative examinations showed that the ratio of the CMAP amplitude on the symptomatic side compared to the asymptomatic side was within the normal range in most patients (58%). In the case of the orbicularis oris muscle, the ratio of the CMAP amplitude on the symptomatic side compared to the asymptomatic side was within the normal range in 50% of patients, whereas it was moderately reduced in the remaining patients.

On the symptomatic side, the standardized latency related to the responses from the orbicularis oculi muscle was within the normal range in 48% of patients, was moderately prolonged in 44% of patients and significantly prolonged in 8% of patients. With regard to responses from the orbicularis oris muscle, the standardized latency was within the normal range in 46% of patients, was slightly prolonged in 52% of patients and significantly prolonged in 2% of patients.

On the asymptomatic side, the CMAP standardized latency recorded from the orbicularis oculi muscle was within the normal range in 52% of patients, while it was slightly prolonged in 48% of patients. With regard to responses from the orbicularis oris muscle, the standardized latency was within the normal range in 70% of patients, was moderately prolonged in 28% of patients and was significantly prolonged in 2% of patients.

Examinations performed 1 month postoperatively related to the responses from both muscles showed that the ratio of the CMAP amplitude on the symptomatic side compared to the asymptomatic side was moderately decreased in most patients (72%) and significantly decreased in 2% of patients.

The standardized latency 1 month postoperatively with regard to responses from the orbicularis oculi muscle and from the orbicularis oris muscle was moderately prolonged in most patients (72% and 74%, respectively). A significant prolongation of the standardized latency was found in 10% of patients in responses from the orbicularis oculi muscle and in 6% of patients in the case of the orbicularis oris muscle.

ENOG examinations performed 6 months postoperatively showed a moderate decrease in the amplitude ratio between symptomatic and asymptomatic sides in 76% of patients with regard to responses from the orbicularis oculi muscle and in 58% of patients in the case of the orbicularis oris muscle. None of the patients presented with a significant decrease in amplitude.

The standardized latency 6 months postoperatively with regard to responses from the orbicularis oculi muscle and from the orbicularis oris muscle was within the normal range in 88% and 82% of patients, respectively.

#### 4.1.2. Blink Reflex Test

Preoperative examinations showed that R1, R2 and R2′ latencies were within the normal range in most patients. Notably, the mean prolongation of the R1 latency was found in 32% of patients, R2 in 28% and R2′ in 32%. Examinations at 1 month after surgery showed the mean prolongation of the latency in most patients (70%, 94% and 94% for R1, R2 and R2′, respectively). The results of another examination at 6 months postoperatively indicated that the mean prolongation of the latency was still present in a significant percentage of patients (Table 3).

### 4.2. Assessment of the Relationship between Postoperative Facial Nerve Paresis and Selected Risk Factors: Predictors of Facial Nerve Paresis after Parotidectomy

An order regression analysis with the enter method was performed to specify electrophysiological and clinical predictors of facial nerve paralysis from the study parameters as well as to create a model that could preoperatively identify patients with a higher complication risk.

The occurrence of FNP was considered a dependent variable. As a result, patients were divided into three groups, i.e., healthy subjects (HB scale grade I), patients with mild/moderate paresis (HB scale grades II and III) and those with moderately severe/severe paresis (HB scale grades IV and V).

The following parameters were introduced as independent variables: parameters of the preoperative ENOG and selected clinical parameters and surgery-related parameters, i.e., sex, histological type of the tumor (mixed tumor, Warthin tumor, other tumors); size of the tumor (<2 cm, 2–4 cm, >4 cm); tumor location (superficial lobe, deep lobe, superficial and deep lobes); type of surgery (superficial parotidectomy, partial superficial parotidectomy, total parotidectomy); intraoperative bleeding (small, average, large); the occurrence of selected symptoms and intraoperative complications (necessary electrocoagulation <1.5 cm from the nerve, wrapped tumor, other complaints, two-fold increase in tumor size within 1 month, diabetes, absorption disorders, stimulants, polyneuropathy); patient’s age; time of surgery and time from tumor diagnosis.

As a result, we created a model that can statistically significantly predict the occurrence of FNP in patients undergoing parotidectomy: χ2(29) = 53.36; *p* = 0.004. A Nagelkerke R2 value of 0.76 means that the model explains 75.8% of the variation of the dependent variable.

Of the variables included in this study, the following predicted the degree of FNP and were statistically significant: Female sex statistically significantly increased the probability of paresis (OR = 59.494 (95% CI: 1.731–2044.877); χ2(1) = 5.13; *p* = 0.024); the histological type of the tumor was associated with the probability of paresis, i.e., both the diagnoses of mixed tumors (OR = 0.006 (95% CI: 0.001–0.336); χ2(1) = 6.21; *p* = 0.013) and Warthin tumors (OR = 0.039 (95% CI: 0.001–0.830); χ2(1) = 4.28; *p* = 0.039) statistically significantly reduced the probability of FNP compared with other types of salivary gland tumors; a tumor size < 2 cm statistically significantly reduced the probability of facial nerve paralysis (OR = 0.003 (95% CI: 0.008–0.800); χ2(1) = 4.16; *p* = 0.041); a lack of complaints in the tumor area statistically significantly reduced the chance of facial nerve paralysis (OR = 0.005 (95% CI: 0.001–0.552); χ2 (1) = 4.87; *p* = 0.027; and a small amount of intraoperative bleeding reduced the probability of FNP compared with an average or large amount of intraoperative bleeding (OR = 0.001 (95% CI: 0.001–0.724); χ2(1) = 4.24; *p* = 0.039; OR = 0.001 (95% CI: 0.001–0.369); χ2(1) = 5.34; *p* = 0.021, respectively).

At the level of statistical tendency, a longer duration of surgery (measured in minutes) was associated with the probability of FNP (OR = 1.037 (95% CI: 0.999–1.077); χ2(1) = 3.61; *p* = 0.058); the type of surgery was associated with the probability of paresis, i.e., partial superficial parotidectomy (OR = 0,002 (95% CI: 0.001–1.178); χ2(1) = 3.65; *p* = 0.056); and superficial parotidectomy (OR = 0.001 (95% CI: 0.001–1.537); χ2(1) = 3.40; *p* = 0.065) reduced the probability of FNP compared with total parotidectomy.

The remaining variables introduced into the study were not statistically significantly related to or associated with FNP at the level of statistical tendency.

The full results of the above analysis presented in the form of a table are included in the Supplementary Materials.

### 4.3. Correlations between Electrophysiological Findings and the Clinical Status of Patients

We decided to verify whether there was a relationship between the electrophysiological results and the clinical status of patients. A number of correlation analyses were performed using the Kendall τ coefficient comparing the results of clinical examinations 1 month after surgery (according to the HB grading scale) and the results of electrophysiological tests shown as the percentage ratio between symptomatic and asymptomatic sides.

The HB score 1 month postoperatively was statistically significantly positively correlated with the standardized latency in the case of the orbicularis oculi muscle, the orbicularis oris muscle and R2 latency 6 months postoperatively. In other words, the higher the values of these parameters in the electrophysiological tests, the higher the HB score was. In addition, a statistically significant negative correlation was found between the HB score 1 month postoperatively and the amplitude of the response from the orbicularis oris muscle in all three examinations. In other words, the lower the values of these parameters in the electrophysiological tests, the higher the HB score was. Notably, the strongest correlation with the clinical examination result was found in the case of the amplitude of the response from the orbicularis oris muscle in the second study (τ = −0.462). There were no significant correlations for the CMAP amplitude. These results may indicate that in our patients a stretch injury of CN 7 was most common.

## 5. Discussion

According to the literature, the prevalence of transient facial nerve dysfunction ranges from 9.3% to 70.2%, whereas permanent facial nerve dysfunction ranges from 0% to 6% [5,6,7,9]. The percentage of patients with transient postoperative FNP in our cohort of patients was higher compared with that of the literature reports. The comparison of the prevalence of postoperative FNP in this study group with the results of other authors is extremely difficult or even impossible. A large discrepancy in the literature data related to the prevalence of postoperative FNP is due to heterogeneous methodology of different studies, i.e., the first postoperative assessment was performed at different time intervals, and the information on paresis was frequently not based on patient examination as reported by retrospective studies; this information came from a history obtained from the patient. The prevalence of paresis was mostly provided collectively without a differentiation of the severity of the dysfunction. Notably, patient groups were mostly heterogeneous as reported by most studies and included patients with different histological tumor types, locations or sizes of lesions who were treated with different procedures. Another reason for discrepancies in the results can be attributed to the specialist who assessed the facial nerve function. In the study group, neurological and clinical assessments of the facial nerve were carried out by the same researcher.

The surgical removal of tumors is often associated with nerve stretching and sometimes with involvement in the vascularization of the nerve [15]. Such situations are often related to a significant risk of neuropraxia in the mechanisms of compression and ischemia, which may lead to FNP or even paralysis. It seems obvious that prolonged surgical time, the coexistence of necrotic tissues and fibrosis may result in postoperative facial nerve dysfunction [2,3,6,7,17].

In our study, among the analyzed risk factors, the following had a statistically significant impact on the occurrence of postoperative FNP: female sex, tumor type, tumor size (>2 cm), small amount of intraoperative bleeding and a lack of preoperative complaints in the tumor area. Type of surgery and a longer duration of surgery were related at the level of statistical tendency.

No correlation was found between the results of electrophysiological tests and the clinical picture in the preoperative assessment. Both ENOG and blink reflex parameters were abnormal in some patients, while none of the subjects presented with FNP (HB scale). The above confirms a higher sensitivity of electrophysiological tests in the diagnosis of facial nerve damage.

A statistically significant negative correlation was found between the grade of FNP on the HB scale 1 month postoperatively and the amplitude of the CMAP response from the orbicularis oris muscle in three examinations (preoperatively and 1 and 6 months after parotidectomy); the strongest correlation with the result of the clinical examination was found for the amplitude of the response from the orbicularis oris muscle 1 month postoperatively (τ = −0.462). A decrease in the amplitude of the response from the orbicularis oris muscle indicates axonal damage in the region of the marginal mandibular branch, which is mostly injured during parotidectomy [18,19].

Notably, preoperative ENOG examinations showed a correlation between the decrease in the amplitude of the response from the orbicularis oris muscle and the increase in FNP assessed 1 month postoperatively. This may indicate the existence of clinically silent axonal changes even preoperatively, probably due to the presence of the tumor. This damage increases due to the influence of intraoperative factors and results in postoperative symptoms of FNP. This correlation has significant clinical implications, since an ENOG examination performed prior to parotidectomy may provide information on clinically silent facial nerve disorders and on the increased risk of postoperative FNP in these patients. In preoperative ENOG examinations, Gao et al. also found a statistically significant decrease in the amplitude in patients with malignant and benign tumors. However, they did not assess the correlation of the study results with the postoperative clinical facial nerve assessment [20]. Aimoni and Bendet presented similar results in patients with salivary gland malignant tumors. This relationship, however, was not found in the case of benign tumors [1,11].

In our group of patients, the statistical analysis showed a correlation between the degree of FNP (assessed based on the HB scale) and the standardized latency of the response from the orbicularis oculi and orbicularis oris muscles 1 month postoperatively, which indicates nerve injury of a demyelinating nature during this period of follow-up. Moreover, the statistical analysis of the blink reflex parameters showed a correlation between the degree of facial nerve injury 1 month postoperatively and the latency of R2 waves 6 months postoperatively.

## 6. Conclusions

To conclude, the factors that may influence the risk of postoperative facial nerve paralysis (prolonged surgical time and the size and location of the tumor other than in the superficial lobe only) may indirectly suggest that surgery-related difficulties and/or surgeon experience can be crucial to the safety of surgery. Furthermore, the preoperative neurophysiological examination of the facial nerve may be useful in planning surgery and predicting surgery-related difficulties.

## Figures and Tables

**Table 1:** Grade of paresis depending on tumor location.

Tumor Location	Grade of Paresis Based on the House–Brackman Scale				
	I	II	III	IV	V
Superficial lobe *n* = 40	12 (30%)	12 (30%)	8 (20%)	6 (15%)	2 (5%)
Deep lobe *n* = 6	1 (16.5%)	3 (50%)	1 (16.5%)	1 (16.5%)	0
Superficial and deep lobes *n* = 4	0	2 (50%)	1 (25%)	1 (50%)	0

**Table 2:** Transient facial nerve paresis depending on type of surgery.

Type of Surgery	Assessment of Paresis Based on the House–Brackman Scale				
	I	II	III	IV	V
Partial superficial parotidectomy	12 (31.5%)	13 (34%)	6 (15.5%)	5 (13%)	2 (5%)
Superficial parotidectomy	1 (16.5%)	1 (16.5%)	3 (50%)	1 (16.5%)	0
Total parotidectomy	0	3 (50%)	1 (16.5%)	2 (33.5%)	0

**Table 3:** Blink reflex: assessment of R1, R2 and R2′ latency.

Examination	Blink Reflex: R1 Latency Symptomatic Side		
	Normal	Prolongation	
		Moderate	Significant
preoperatively	34 (68%)	16 (32%)	0
1 month postoperatively	15 (30%)	35 (70%)	0
6 months postoperatively	26 (52%)	24 (48%)	0
Examination	Blink Reflex: R2 Latency Symptomatic Side		
	Normal	Prolongation	
		Moderate	Significant
preoperative	36 (72%)	14 (28%)	0
postoperative at 1 month	1 (2%)	47 (94%)	2 (4%)
postoperative at 6 months	4 (8%)	46 (92%)	0
Examination	Blink Reflex: R2′ Latency Asymptomatic Side		
	Normal	Prolongation	
		Moderate	Significant
preoperative	34 (68%)	16 (32%)	0
postoperative at 1 month	1 (2%)	47 (94%)	2 (4%)
postoperative at 6 months	6 (12%)	44 (88%)	0

## Data Availability

The authors have already attached supplementary data.

## Supplementary Materials

The following supporting information can be downloaded at: https://www.mdpi.com/article/10.3390/medicina58121726/s1.
