# Supplementary material for: Prospective Assessment of Risk Factors Influencing Facial Nerve Paresis in Patients after Surgery for Parotid Gland Tumors
Source: Medicina (Kaunas). 2022 Nov 25;58(12):1726. doi: 10.3390/medicina58121726 (PMC9781186; doi:10.3390/medicina58121726)
Supplement: Supplementary file 1 [file medicina-58-01726-s001.zip › medicina-1903602-supplementary.pdf]

Table S1. Assessment of the Relationship between Postoperative Facial Nerve Paresis and Selected Risk Factors. An order regression analysis.

							95% CI for B			***95% CI for OR***	
		B	***SE***	***Wald***	***df***	***p***	***LL***	***UL***	***OR***	***LL***	***UL***
Threshold	[degree of paresis = 1,00]	-5,90	7,05	0,70	1	0,403	-19,71	7,92	0,003	0,00	2741,68
	[degree of paresis = 2,00]	0,52	6,66	0,01	1	0,938	-12,53	13,57	1,675	0,00	779256,45
Location	age years	0,03	0,05	0,40	1	0,527	-0,07	0,14	1,034	0,93	1,15
	duration of surgery min	0,04	0,02	3,61	1	**0,058**	0,00	0,08	1,037	1,00	1,08
	Time from tumor diagnosis months	0,01	0,01	0,44	1	0,510	-0,02	0,04	1,009	0,98	1,04
	OCULI amplitude sympt/asympt 1 exam	-2,76	2,46	1,26	1	0,262	-7,59	2,07	0,063	0,00	7,88
	OCULI stand latency sympt/asympt 1 exam	1,90	1,40	1,86	1	0,172	-0,83	4,64	6,714	0,44	103,52
	ORIS amplitude sympt/asympt 1 exam	-2,61	2,47	1,12	1	0,291	-7,45	2,23	0,073	0,00	9,32
	[sex=1,00]	4,09	1,81	5,13	1	**0,024**	0,55	7,62	59,494	1,73	2044,88
	[histological type=1,00]	-5,11	2,05	6,21	1	**0,013**	-9,12	-1,09	0,006	0,00	,34
	[histological type =2,00]	-3,54	1,71	4,28	1	**0,039**	-6,90	-0,19	0,029	0,00	,83
	[size of the tumor=1,00]	-5,66	2,78	4,16	1	**0,041**	-11,10	-0,22	0,003	0,00	,80
	[size of the tumor=2,00]	-2,60	1,90	1,87	1	0,172	-6,32	1,13	0,075	0,00	3,08
	[tumor location=1,00]	8,08	3,70	4,78	1	**0,029**	0,84	15,32	3230,969	2,31	4515261,58
	[tumor location=2,00]	4,97	3,19	2,43	1	0,119	-1,28	11,22	143,772	0,28	74644,02
	[type of parotidectomy=1,00]	-6,36	3,33	3,65	1	**0,056**	-12,88	0,16	0,002	0,00	1,18
	[type of parotidectomy=2,00]	-6,89	3,73	3,40	1	**0,065**	-14,21	0,43	0,001	0,00	1,54
	[intraoperative bleeding=1,00]	-6,68	3,24	4,24	1	**0,039**	-13,04	-0,32	0,001	0,00	,72
	[intraoperative bleeding=2,00]	-6,58	2,85	5,34	1	**0,021**	-12,16	-1,00	0,001	0,00	,37
	[electrocoagulation <1.5 cm from the nerve=1,00]	0,12	1,35	0,01	1	0,930	-2,54	2,77	1,126	0,08	15,99
	[wrapped tumor=1,00]	0,19	1,52	0,02	1	0,901	-2,79	3,17	1,208	0,06	23,75
	[other complaints=1,00]	-5,34	2,42	4,87	1	**0,027**	-10,09	-0,60	0,005	0,00	,55
	[2-fold increase in tumor size in 1 month=1,00]	-1,32	3,10	0,18	1	0,670	-7,39	4,75	0,267	0,00	115,49
	[diabetes=1,00]	-1,03	1,93	0,28	1	0,596	-4,81	2,76	0,359	0,01	15,81
	[malabsorption=1,00]	-0,96	3,16	0,09	1	0,762	-7,16	5,24	0,383	0,00	188,69
	[stimulants=1,00]	3,40	2,53	1,81	1	0,178	-1,55	8,36	30,058	0,21	4253,01
